# Association of the bleeding time test with aspects of traumatic brain injury in patients with alcohol use disorder

**DOI:** 10.1007/s00701-020-04373-y

**Published:** 2020-05-18

**Authors:** P. P. Tsitsopoulos, N. Marklund, E. Rostami, P. Enblad, L. Hillered

**Affiliations:** 1 Department of Neuroscience, Section of Neurosurgery, Uppsala University, Uppsala, Sweden; 2 Department of Clinical Sciences Lund, Neurosurgery, Skåne University Hospital, Lund University, Lund, Sweden

**Keywords:** Traumatic brain injury, Injury severity, Alcohol use disorder, Hemostasis, Platelets, IVY bleeding time test, Outcome

## Abstract

**Background—aim:**

Traumatic brain injury (TBI) and alcohol use disorder (AUD) can occur concomitantly and be associated with coagulopathy that influences TBI outcome. The use of bleeding time tests in TBI management is controversial. We hypothesized that in TBI patients with AUD, a prolonged bleeding time is associated with more severe injury and poor outcome.

**Material and methods:**

Moderate and severe TBI patients with evidence of AUD were examined with bleeding time according to IVY bleeding time on admission during neurointensive care. Baseline clinical and radiological characteristics were recorded. A standardized IVY bleeding time test was determined by staff trained in the procedure. Bleeding time test results were divided into normal (≤ 600 s), prolonged (> 600 s), and markedly prolonged (≥ 900 s). Normal platelet count (PLT) was defined as > 150,000/μL. This cohort was compared with another group of TBI patients without evidence of AUD.

**Results:**

Fifty-two patients with TBI and AUD were identified, and 121 TBI patients without any history of AUD were used as controls. PLT was low in 44.2% and bleeding time was prolonged in 69.2% of patients. Bleeding time values negatively correlated with PLT (*p* < 0.05). TBI patients with markedly prolonged values (≥ 900 s) had significantly increased hematoma size, and more frequently required intracranial pressure measurement and mechanical ventilation compared with those with bleeding times < 900 s (*p* < 0.05). Most patients (88%) with low platelet count had prolonged bleeding time. No difference in 6-month outcome between the bleeding time groups was observed (*p* > 0.05). Subjects with TBI and no evidence for AUD had lower bleeding time values and higher platelet count compared with those with TBI and history of AUD (*p* < 0.05).

**Conclusions:**

Although differences in the bleeding time values between TBI cohorts exist and prolonged values may be seen even in patients with normal platelet count, the bleeding test is a marker of primary hemostasis and platelet function with low specificity. However, it may provide an additional assessment in the interpretation of the overall status of TBI patients with AUD. Therefore, the bleeding time test should only be used in combination with the patient’s bleeding history and careful assessment of other hematologic parameters.

## Introduction

Traumatic brain injury (TBI) is frequently associated with coagulation abnormalities which may negatively influence outcome [12, 27, 32, 43, 46, 51]. The underlying mechanisms leading to hemostatic problems following TBI are complex involving factors which can either separately or synergistically impair components of the coagulation system leading to platelet dysfunction and increased fibrinolysis [14, 23, 32, 42, 51, 57]. Although significant progress has been made in recent years, the detailed pathophysiology of coagulation pathway disturbance following TBI remains poorly understood [23, 57].

Alcohol consumption has by itself been shown to influence hemostasis [3, 30, 41]. In healthy individuals, platelet aggregation and bleeding time are impaired immediately following alcohol intake [8], related to the amount of ingested alcohol [30]. Following TBI, acute and chronic alcohol use may predispose to hemorrhage by influencing coagulation and fibrinolytic systems which may in turn exacerbate outcome [21, 36].

Methods for measuring bleeding time in vivo have been applied in clinical practice with the aim to recognize problems of primary hemostasis since its first introduction in 1910 [6]. In later decades, the cutaneous “venostasis” bleeding time technique was modified, standardized, and popularized by Ivy and others [2, 17, 18, 22]. The most common indications for the application of the IVY bleeding time test is the preoperative investigation of platelet function and primary hemostasis [4, 9, 13, 39]. Due to poor sensitivity and specificity and standardization problems of the IVY test, there is controversy regarding its efficacy to predict bleeding risk [4, 9, 24, 37]. However, it remains in use in many clinical practices including our neurosurgical department after the implementation of a carefully standardized testing protocol [28, 32, 38, 48]. It should also be noted that studies reporting controversial results regarding the IVY method did not include neurosurgical patient cohorts [4, 24, 37].

In this study, we tested the hypothesis that in patients with alcohol use disorder (AUD) and moderate or severe TBI, a prolonged bleeding time assessed by IVY is associated with injury progression and poor outcome and provides any added clinical value over platelet count.

## Materials and methods

### Patients and setting

The study was retrospectively performed in the Department of Neurosurgery at Uppsala University Hospital, Uppsala, Sweden, where the IVY bleeding time test is routinely used in neurosurgical patients with a clinical suspicion of coagulopathy. For inclusion, the patients needed to meet the following criteria: (1) moderate TBI (Glasgow Coma Scale (GCS) scores 9–13) or severe TBI (GCS ≤ 8), (2) admitted to the neurointensive care unit (NICU) from January 2008 to December 2016, (3) ≥ 18 years old, (4) indications of known history of chronic alcohol abuse from the patients’ records and ICD codes, (5) bleeding time according to the IVY test performed. The exclusion criteria were the following: (1) multitrauma that may have influenced coagulation mechanisms (Injury Severity Score [ISS] > 16), (2) marked preexisting or observed coagulation abnormalities such as international normalized ratio (INR) > 1.4 and partial thromboplastin time (PTT) > 42 s and innate problems such as hemophilia and von Willebrand disease, (3) patients on antiplatelet medication. Subjects with marked liver dysfunction reflected by prolonged hematologic parameters and liver function tests as well as bone marrow dysfunction were also not included in our study sample. The reference rates for activated partial thromboplastin time (aPTT) in our laboratory are 26–42 s. A control group with data from 121 patients from 2008 to 2012 including other TBI patients evaluated by the IVY test was also included and compared. Multitrauma cases with marked coagulation disturbances (such as hemophilia and von Willebrand disease), those on antiplatelet medication, patients with preexisting significant coagulopathies, and patients with AUD were excluded. A small number of patients were on anticoagulants (*n* = 6) but those with INR > 1.4 and PTT > 42 s were not included in the cohort.

Baseline clinical and radiological characteristics were recorded and compared by an evaluator blinded to the bleeding time tests. The level of consciousness on admission and at deterioration (if any) was evaluated with the use of the GCS score [47]. Deterioration was defined as any worsening in the neurological status during the initial period of hospitalization. All patients underwent repeat computed tomography (CT) scans of the head, determined on individual clinical decisions. The CT scan findings classified the injury as focal or diffuse based on their dominant radiological characteristics [31]. CT scans were also screened during hospitalization to determine possible increase in hematoma size, midline shift, and ventricle size [31]. Changes in hematoma size (increase or decrease) during hospitalization were determined based on the radiology report and personal observations. Any increase in the midline shift was defined as the displacement of the septum pellucidum in relation to the midline in millimeters [19]. Surgical data with an emphasis on the need for decompressive surgery (hematoma removal and/or craniectomy) or non-decompressive surgery (intraparenchymal or intraventricular measurement of intracranial pressure) were also documented. Information on the history of AUD was extracted from the patient archives.

### Bleeding time

Bleeding time was calculated using the IVY bleeding time test on admission. The bleeding time test is used routinely in TBI patients with suspected or confirmed bleeding disorder and in individuals on antiplatelet/anticoagulant drugs who are admitted to the NICU according to a standardized protocol.

The Surgicutt^©^ device which represents a refinement and a modification of the IVY bleeding test was used [15]. During testing, a blood pressure cuff is applied on the upper arm and inflated to 50 mmHg to control capillary tone, maintain constant capillary pressure, and better standardize the procedure. Next, a sterile blood lancet performs a shallow intradermal incision over the bend of the supinated forearm. Every 30 s, a filter paper is used to gently absorb the blood and check for hemostasis. The time period from skin incision until the bleeding has stopped is measured. In our laboratory, regardless of the underlying diagnosis and pathology, and in the general population, values < 600 s are considered normal, values of 600–900 s prolonged, and values ≥ 900 s markedly prolonged which is also the time point when the examiner stops the testing. Thus, no exact value > 900 s is recorded, and thus, we used ≥ 900 s in our categorization of the patient cohort.

Testing was done by experienced nurse assistants well familiar with this procedure. For quality control of the bleeding time test, our full-time NICU lab technician teaches the participating nurse assistants how to perform the test and issues a permit to perform bleeding time on patients after demonstration of the technique. The number of tests for each nurse assistant is continuously monitored. If the annual test rate falls low for any individual nurse assistant or is otherwise needed, a brushup session with the NICU technician is offered using an instruction video. All test results are continuously monitored by the responsible technician to check for aberrant data clusters for each nurse assistant.

### Platelet count and other coagulation tests

The number of platelets was recorded on admission as a part of the blood count analysis. Other routine coagulation parameters such as PTT, prothrombin time (PT), and INR were also documented. Testing was done at the accredited laboratory of the Department of Clinical Chemistry, at Uppsala University Hospital.

After diagnosis, coagulation problems were corrected with platelet transfusions and/or a combination of plasma transfusion, desmopressin, and tranexamic acid, if needed. None of the included patients was given prothrombin complex concentration on admission since those with prolonged INR (> 1.4) were excluded from the analysis.

### Neurointensive care

The standardized protocol in our neurointensive care unit is based on intensive physiological monitoring and therapy of any derangement to avoid or minimize secondary brain injury. An intracranial pressure (ICP) and cerebral perfusion pressure (CPP)–guided protocol was applied. This included slight hyperventilation/normoventilation, head elevation (30°), and careful volume expansion to obtain normovolemia. Extracerebral hematomas and contusions with significant mass effect were surgically evacuated according to the treating physician’s judgment. Unconscious patients were mechanically ventilated with monitoring of the ICP. ICP was monitored in unconscious patients with either intraventricular drainage catheter or intraparenchymal probes. Decompressive craniectomy was the last-tier treatment choice. A combination of intermittent intravenous morphine and continuous intravenous propofol infusion was given for analgesia and sedation. Arterial blood gases and blood glucose levels were also monitored. The treatment goals were an ICP of ≤ 20 mmHg and a CPP of ≥ 60 mmHg [7]. In case of substantially prolonged IVY bleeding time, no ICP device was initially inserted and frequent wake-up tests were performed to detect any deterioration [29]. In these patients, we aimed to correct the coagulation abnormalities, and an ICP device was typically inserted after normalization of the bleeding time.

According to the standardized procedures at the neurointensive care unit, the core temperature was kept below 38 °C. The recommended treatment of hyperthermia was acetaminophen, then cooling blankets, and finally infusions of thorazine. To reduce physiological stress response, infusion of 0.2–0.3 mg/kg/24 h β1-antagonist metoprolol was given and injections of α2-agonist clonidin (0.5–1.0 μg/kg × 8 or the same dose as an infusion) [34, 53].

### Outcome measures

Τhe cohort was categorized into IVY ≤ 600 s (normal), > 600 (prolonged), and ≥ 900 s (markedly prolonged, the time point where testing is discontinued). PLT values > 150,000/μL were considered normal whereas values < 150,000/μL were considered below normal (low) [10, 11].

Patients were followed up until discharge from the neurosurgery department and at 6 months. Neurological outcome was assessed at 6 months with the extended Glasgow Outcome Scale (GOS-E) [55].

### Statistical analysis

Data entry was performed using Microsoft Excel (Microsoft Corp., Redmond, WA, USA). Normality of data was evaluated with the Kolmogorov–Smirnov test. Continuous variables were evaluated for statistical significance using Student’s *t* test or Mann–Whitney *U*, as appropriate. Categorical variables were evaluated for statistical significance using Fisher’s exact test, as appropriate. Correlations between tested values were calculated using linear regression. The following comparisons were done:

1. Bleeding time > 600 s vs. bleeding time ≤ 600 s
2. Bleeding time ≥ 900 s vs. bleeding time < 900 s
3. PLT > 150,000/μL vs. PLT < 150,000/μL

Analysis was done using the statistical software SPSS version 22 (IBM, New York, NY, USA) and GraphPad Prism 7 (GraphPad Inc., San Diego, CA). *p* values below 0.05 were considered statistically significant.

## Results

### Baseline characteristics

**Fig. 1:**
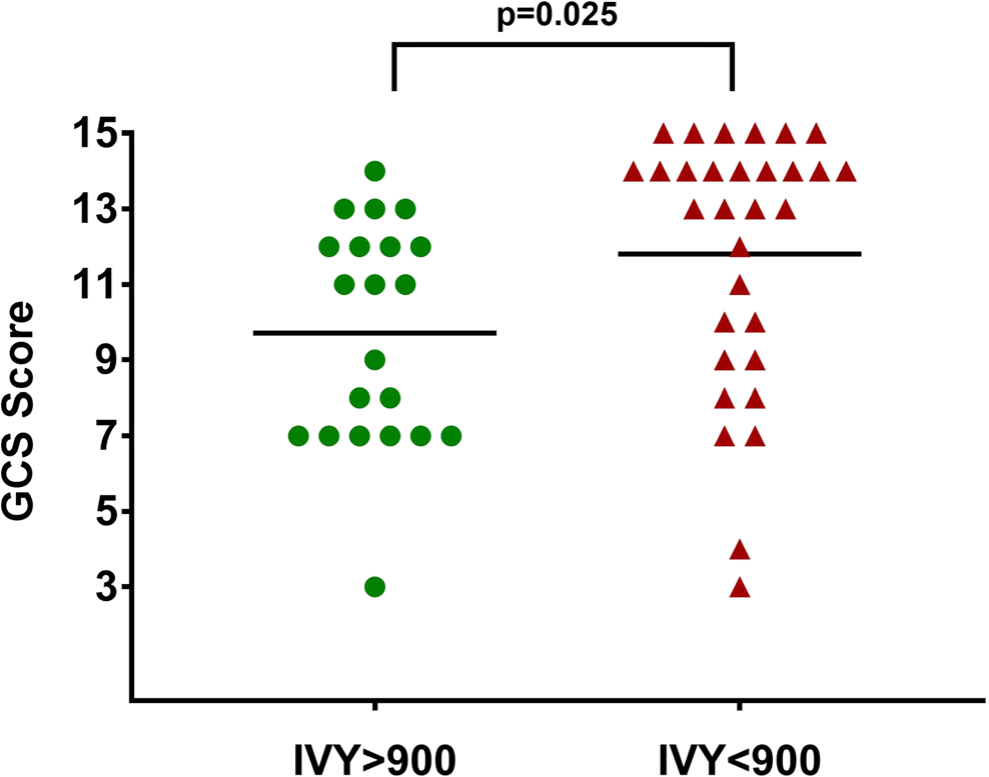
Graph showing Glasgow Coma Scale (GCS) scores at discharge from the NICU in patients with markedly prolonged (≥ 900 s) and normal or prolonged (< 900 s) IVY values (*p* = 0.025)

During the study period, 52 patients met the inclusion criteria. Patients’ characteristics are shown in Table 1. Their mean age was 58 ± 11 (range 25–74) years, and the mean length of stay in the neurointensive care unit was 10 ± 7 days. Most TBIs (88.4%) resulted from falls. PLT was below normal values in 44.2% and bleeding time was prolonged in 69.2% of patients. No patient was on antiplatelet medication and only two were on anticoagulation. Of the 84.6% patients that underwent any type of neurosurgery, 48% required a decompressive operation (removal of hematoma and/or decompressive craniectomy). Increase in hematoma volume from the initial CT scan during hospitalization was seen in 65.3%. GCS scores at discharge in patients with IVY < 900 s were significantly higher compared to those with IVY≥900 s (p=0.025; Fig. 1). Six month outcome was available for 43/52 patients (82.7%; Table 1). At the 6-month follow-up, 8/43 (19%) of patients in which the outcome was evaluated were dead.

**Table 1:** Baseline characteristics of 52 patients with alcohol use disorder and moderate or severe traumatic brain injury. Values are presented as mean and standard deviation

Characteristic	*N*	%
*N* of patients	52	
Age (years)	58 ± 11	
Length of stay (days)	13 ± 7.4	
ICU stay (days)	10 ± 7	
ICP monitoring	33	63.4
Multitrauma (ISS > 16)	3	5.7
Cause of injury		
Fall	46	88.4
Road accident	4	7.6
Other	2	3.8
Use of anticoagulation*	2	3.8
Focal intracranial pathology	29	55.8
Multifocal intracranial pathology	23	44.2
Abnormal PLT (< 150,000/μL)	23	44.2
INR 1.3-1.4	4	7.6
a PTT 36-42 s	7	13.4
Surgery	44	84.6
ICP measurement	33	75
Decompressive surgery	25	48
Reoperation	22	50
Decompressive surgery	14	31.8
Ventilator	46	88.4
Number of CT scans	5.5 ± 2.5	
Increased midline shift	26	50
Increased hematoma size	34	65.3

*CT*, computed tomography; *GCS*, Glasgow Coma Scale; *GOSE*, extended Glasgow Outcome Scale; *ICP*, intracranial pressure monitoring; *ICU*, neurointensive care unit; *ISS*, Injury Severity Score; *INR*, international normalized ratio; *PLT*, platelet count; *PTT*, partial thromboplastin time

*Warfarin

### Bleeding time

Thirty-three patients (63.4%) had prolonged bleeding time (> 600 s). In those individuals, increased midline shift (60.1% vs. 26.3%) and hematoma size (78.8% vs. 36.9%) on repeat CT scans were noted compared with those with normal values (≤ 600 s; *p* < 0.05). The frequency for decompressive surgery was similar among the groups (*p* > 0.05; Table 2).

**Table 2:** Bleeding time > 600 s vs. bleeding time < 600 s in the patients with traumatic brain injury and alcohol use disorder. Values are presented as mean and standard deviation or median and average

Characteristic	Bleeding time > 600 s	Bleeding time < 600 s	*p* value
*N* of patients (*n* = 52)	33 (63.4%)	19 (36.5%)	
Age (years)	55.9 ± 10.3	60.8 ± 11.6	0.12
Length of stay (days)	14.2 ± 8.2	11.2 ± 5.2	0.15
ICU stay (days)	11.1 ± 7.4	8.4 ± 6.1	0.18
ICP measurement	25 (75.8%)	10 (52.6%)	0.13
Multitrauma (ISS > 16)	2 (6%)	1 (5.2%)	1.00
Focal intracranial pathology	17 (51.5%)	12 (63.1%)	0.56
Multifocal intracranial pathology	16 (48.5%)	7 (36.9%)	0.56
Abnormal PLT (< 150,000/μL)	18 (54.5%)	5 (26.3%)	0.08
Surgery	29 (87.9%)	15 (78.9%)	0.44
ICP only	11 (33.3%)	7 (36.9%)	1.00
Decompressive surgery	18 (54.5%)	8 (42.1%)	0.75
Reoperation	15 (45.4%)	7 (36.9%)	1.00
Decompressive surgery	8 (24.2%)	6 (31.6%)	0.39
Ventilator	30 (90.1%)	16 (84.2%)	0.65
Number of CT scans	5.6 ± 2.6	5.2 ± 2.3	0.51
Increased midline shift	20 (60.1%)	5 (26.3%)	0.02*
Increased hematoma size	26 (78.8%)	7 (36.9%)	0.006*
GCS score on admission	10 (3–14)	9 (5–14)	0.53
GOSE score at 6 months (good/bad)	11/16	5/11	0.74

*CT*, computed tomography; *GCS*, Glasgow Coma Scale; *GOSE*, extended Glasgow Outcome Scale; *ICP*, intracranial pressure monitoring; *ICU*, intensive care unit; *ISS*, Injury Severity Score; *PLT*, platelet count

*Statistical significance

Patients with markedly prolonged bleeding time initially (≥ 900 s, 40.4%) had more frequently ICP monitoring compared with those with bleeding time < 900 s (*p* < 0.05). All patients with bleeding time ≥ 900 s had mechanical ventilation. A larger proportion of patients with markedly prolonged values had increased hematoma size as seen on repeat CT scans compared with those with normal of slightly prolonged values (80.9% vs. 50.6%, *p* < 0.05) (Table 3; *p* < 0.05).

**Table 3:** Bleeding time ≥ 900 s vs. bleeding time < 900 s in patients with traumatic brain injury and alcohol use disorder. Values are presented as mean and standard deviation or median and average. 900 s is the time point where the test stops

Characteristic	Bleeding time ≥ 900 s	Bleeding time < 900 s	*p* value
*N* of patients (*n* = 52)	21 (40.4%)	31 (59.6%)	
Age (years)	55.8 ± 9	58.9 ± 12.1	0.31
Length of stay (days)	14.3 ± 8.5	12.3 ± 6.5	0.33
ICU stay (days)	12.2 ± 6.1	8.6 ± 7.3	0.07
ICP monitoring	19 (90.5%)	14 (45.2%)	0.001*
Multitrauma (ISS > 16)	1 (4.7%)	2 (6.4%)	1.00
Focal intracranial pathology	10 (47.6%)	19 (61.3%)	0.39
Multifocal intracranial pathology	11 (52.4%)	12 (38.7%)	0.39
Abnormal PLT (< 150,000/μL)	12 (57.1%)	11 (35.5%)	0.16
Surgery	20 (95.2%)	24 (77.4%)	0.12
ICP only	7 (33.3%)	11 (35.5%)	1.00
Decompressive surgery	14 (66.7%)	13 (41.9%)	0.35
Reoperation	11 (52.4%)	11 (35.5%)	0.76
Decompressive surgery	6 (28.6%)	7 (22.6%)	1.00
Ventilator	21 (100%)	18 (58.0%)	0.02*
Number of CT scans	6.1 ± 2.9	5 ± 2.1	0.13
Increase in midline shift	13 (61.2%)	12 (38.7%)	0.15
Increase of hematoma size	17 (80.9)	16 (51.6%)	0.04*
GCS score on admission	10 (3–14)	9 (5–14)	0.66
GOSE score at 6 months (good/bad)	6/10	10/17	1.00

*CT*, computed tomography; *GCS*, Glasgow Coma Scale; *GOSE*, extended Glasgow Outcome Scale; *ICP*, intracranial pressure monitoring; *ICU*, intensive care unit; *ISS*, Injury Severity Score; *PLT*, platelet count

*Statistical significance

Compared with those in the control group (*n* = 121), overall, prolonged bleeding time values were found in TBI patients with history of AUD (Table 4; *p* < 0.05).

**Table 4:** Comparison of bleeding time values and platelet count in patients with TBI and AUD with those with TBI without AUD

	Age (years)	Bleeding time > 600 s	Bleeding time > 900 s	PLT < 150,000/μL	PLT < 100,000/μL
TBI-AUD group	57.3 ± 10.8 (25–74)	33/52	21/52	25/52	11/52
TBI-control group	56.4 ± 18.4 (18–86)	37/121	15/121	34/121	7/121
*p* value	> 0.05	< 0.001*	< 0.001*	0.014*	0.005*

*AUD*, alcohol use disorder; *TBI*, traumatic brain injury

*Statistical significance

**Fig. 2:**
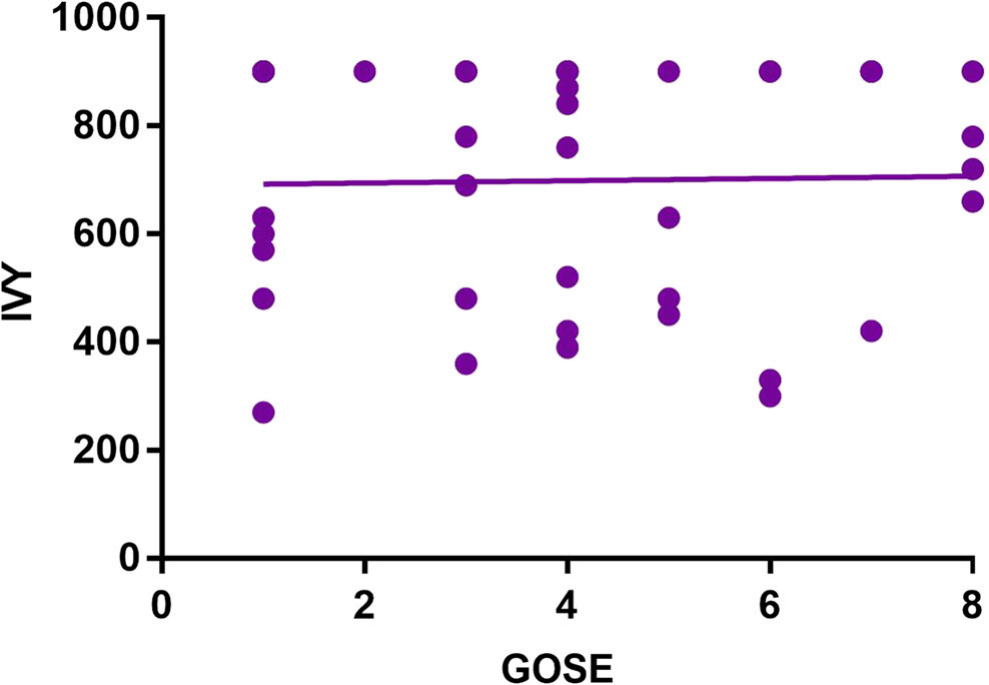
Regression analysis showing the relationship of IVY bleeding time values with 6-month outcome in the current cohort (Spearman *r* = − 0.010; *p* = 0.948)

No difference in 6-month outcome was observed when normal and prolonged bleeding time values were compared (Tables 2 and 3; *p* > 0.05, Fig. 2).

### Platelet count

**Fig. 3:**
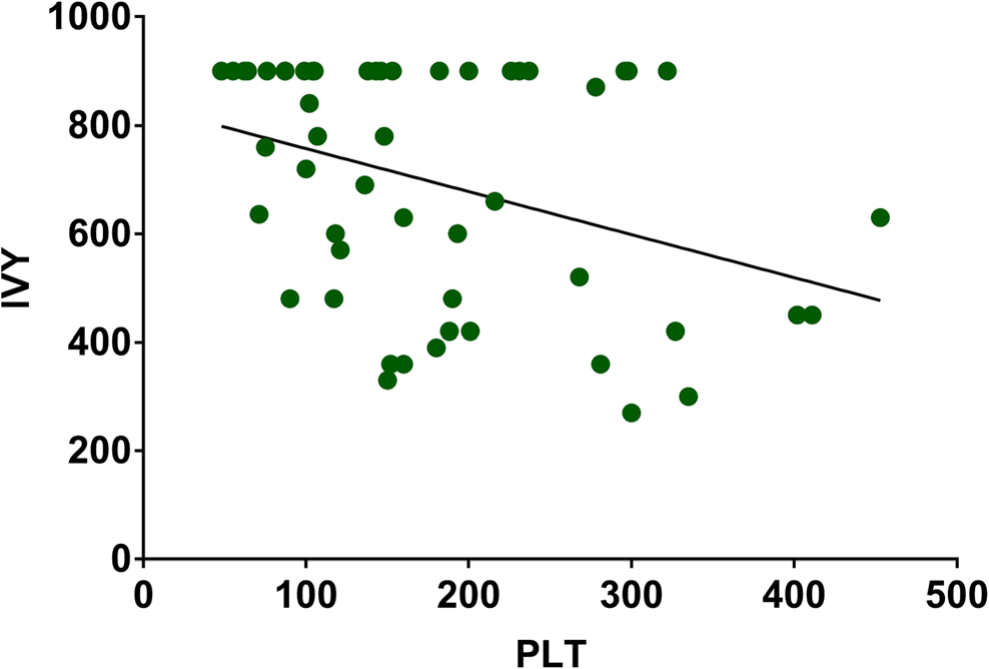
Regression analysis showing the relationship between IVY bleeding time values and platelet count in the current cohort. Note the negative correlation between the two parameters (Spearman *r* = − 0.369; *p* = 0.007)

IVY values negatively correlated with PLT (*p* < 0.05; Fig. 3). More patients with platelet count < 150,000/μL had prolonged IVY results compared with those with normal platelet count (88% vs. 51.9%; *p* < 0.05). No differences were seen between the groups (PLT > 150,000/μL vs. PLT < 150,000/μL) in the need for surgery (decompressive or not) and radiological parameters (*p* > 0.05; Table 5).

**Table 5:** Normal PLT (> 150,000/μL) vs. abnormal PLT (< 150,000/μL) in patients with traumatic brain injury and alcohol use disorder. Values are presented as mean and standard deviation or median and average

Characteristic	PLT > 150,000 /μL	PLT < 150,000 /μL	*p* value
*N* of patients (*n* = 52)	27 (51.9%)	25 (48.1%)	
Age (years)	57.4 ± 12.8	58 ± 8.9	0.83
Length of stay (days)	12.4 ± 7.2	13.9 ± 7.6	0.47
ICU stay (days)	9.18 ± 7.4	11 ± 6.5	0.34
ICP monitoring	16 (59.2%)	17 (68.0%)	0.57
Multitrauma (ISS > 16)	2 (7.4%)	1 (4.0%)	1.00
Focal intracranial pathology	14 (51.9%)	15 (60.0%)	0.58
Multifocal intracranial pathology	13 (48.1%)	10 (40.0%)	0.58
Abnormal bleeding time (> 600 s)	14 (51.9%)	22 (88.0%)	0.006*
Bleeding time > 900 s	8 (29.6%)	13 (52.0%)	0.15
Surgery	23 (85.2%)	21 (84.0%)	1.00
ICP	8 (29.6%)	10 (40.0%)	0.56
Decompressive surgery	16 (59.3%)	11 (44.0%)	0.37
Reoperation	11 (40.7%)	11 (44.0%)	0.16
Decompressive surgery	7 (25.9%)	6 (24.0%)	1.00
Ventilator	22 (81.4%)	24 (96.0)	0.19
Number of CT scans	5.1 ± 2.8	5.8 ± 2.1	0.29
Increase in midline shift	10 (37.0%)	15 (60.0%)	0.16
Increase of hematoma size	15 (55.5%)	18 (72.0%)	0.25
GCS score on admission	9 (5–14)	9 (3–14)	0.29
GOSE score at 6 months (good/bad)	8/15	8/12	0.76

*CT*, computed tomography; *GCS*, Glasgow Coma Scale; *GOSE*, extended Glasgow Outcome Scale; *ICP*, intracranial pressure monitoring; *ICU*, intensive care unit; *ISS*, Injury Severity Score; *PLT*, platelet count

*Statistical significance

TBI subjects with a history of AUD had lower platelet count compared with those without a history of AUD (Table 4; *p* < 0.05).

No difference in 6-month outcome was seen when patients with normal platelet count > 150,000/μL were compared with those with < 150,000/μL (Table 5; *p* > 0.05).

## Discussion

This study tested the hypothesis that in moderate-to-severe TBI patients with alcohol use disorder (AUD), a prolonged bleeding time is associated with injury progression and poor outcome. IVY bleeding time values were investigated independently and in correlation with platelet count. As expected, values negatively associated with platelet count. Patients with markedly prolonged bleeding time had a larger increase in hematoma size and required more often ICP monitoring and mechanical ventilation. TBI subjects with no evidence of AUD had lower bleeding time values and higher platelet count compared with those with evidence of AUD. Neither bleeding time nor platelet count correlated with 6-month outcome.

The incidence, type, and extent of coagulopathy in TBI during hospitalization are variable [12, 23, 27, 32]. Due to coagulopathy, preexisting lesions can progress and new lesions may appear [23, 44, 45]. Alcohol consumption can influence coagulation in a complex way causing thrombocytopenia, impaired platelet function, and diminished fibrinolysis [1, 32, 49]. A negative correlation between blood alcohol concentration and GCS scores was found in patients with low Rotterdam CT scores [40]. However, in the same study, no difference was noted in outcome between alcohol-negative and alcohol-positive patients [40].

Some advantages of the IVY bleeding test include its ability to evaluate natural primary hemostasis in the vessel wall as an indicator of platelet function, the low cost, its accessibility, and its minimally invasive features [13, 33]. However, its relevance has been repeatedly questioned in recent years. Numerous reports have stated that this test does not reflect hemostasis in other areas, it is poorly reproducible, and there are difficulties in standardization; it may be normal in patients with known bleeding tendency as well as in patients on antiplatelet medication, or abnormal in individuals with a negative history of coagulation problems [13, 20, 24, 52]. Moreover, it can be time consuming for the staff conducting the test. It has also been stated that even normal bleeding time values cannot reliably exclude patients prone to bleeding in surgery, emphasizing the importance of a careful and detailed bleeding history [5, 24, 37]. Nevertheless, mainly due to its simplicity and natural results, there are centers still using the bleeding test as a screening tool in patients prone to bleeding either preoperatively or during hospitalization, especially when more specialized tests are unavailable, expensive, or time consuming. Moreover, there is paucity of data on the use of the bleeding test in neurosurgical patients.

Commercially available devices such as the Simplate^®^ and the Surgicutt^®^ methods are continued refinements and modifications of the IVY bleeding test [15]. The Surgicutt^®^ device is a method which is also used in our department. Regarding the Simplate^®^ device, it has not been confirmed to be superior in sensitivity or reproducibility to the IVY method, which is cheaper, takes less time, and does not leave scars [22]. It has also been proven that the Surgicutt^®^ and the Simplate^®^ devices have been comparable in many aspects but a horizontal Surgicutt^®^ bleeding time is likely most sensitive for the detection of primary hemostasis [2].

In the current study, special emphasis was placed on the importance of the IVY bleeding test performed by well-trained and experienced nurse assistants, and how it correlates with platelet count, progression of the initial injury, and patient outcome. In patients where INR and PTT values were prolonged during testing, these were slightly abnormal (INR < 1.5, PTT < 42 s), implying that their influence on the present observations was non-significant compared with the effect of the bleeding time and the number of platelets.

The clinical course during neurointensive care treatment differed in some aspects between patients with prolonged bleeding time (> 600 s, 64.7%) and those with normal values (≤ 600 s, 36.5%). More patients with prolonged bleeding time had an increase in hematoma size and in the midline shift as evidenced on repeat CT scans. It remains unknown whether this increase resulted from prolonged bleeding time values or from the natural course and progression of intracranial hematoma [35]. No difference in the number of patients that required decompressive surgery or reoperation was seen, and outcome at discharge did not differ between the two groups. It should be noted that in patients with coagulation problems including prolonged bleeding time, special effort was made to correct them as soon as possible. Therefore, it may be concluded that moderately prolonged values do not significantly impact the outcome in chronic alcohol abusers from the surgical and clinical TBI perspective, given the condition that prolonged values are normalized shortly following their detection.

In individuals with markedly prolonged bleeding time values (≥ 900 s, 40.3%), the impact on injury severity was more evident. In addition to an increase in hematoma size, more patients required ICP monitoring and mechanical ventilation. Regarding ICP monitoring in patients with severe coagulation problems, insertion of ICP catheter is postponed until the coagulation status is sufficiently improved. However, in the current cohort, this was only partially true since only 28.6% of patients with bleeding time > 900 s received ICP monitoring later than 24 h from injury. When the two groups were compared, no difference either in the need for decompressive surgery or reoperation was noted. More patients with thrombocytopenia were included in the > 900s bleeding time group, but this difference was not statistically significant. Therefore, it can be postulated that bleeding time may, to some extent, behave differently compared with platelet count. It should be noted though that no difference in the 6-month outcome was seen between the groups.

When patients with normal platelet count (51.9%) were compared with those with low platelet count (48.1%), abnormal bleeding time values were noted in most patients with thrombocytopenia (88%), as expected. However, when these groups were compared, no difference was seen in patients with markedly prolonged bleeding time values (≥ 900 s), increase in hematoma size, and need for surgery. It should be noted that in patients with thrombocytopenia compared with those with normal platelet count, the 6-month outcome was similar. In TBI, the platelet count below 100,000/μL is associated with increased mortality and is a commonly used cutoff in neurosurgery [25]. However, the present study investigated the possible added value of IVY in those patients where coagulation disorders were not obvious and chose the reference value of 150,000/μL as our cutoff based on the existing laboratory reference from the literature [10, 11]. Noticeably, only one patient had platelet count below 50,000/μL and in total, 11/52 had platelet count below 100,000/μL, representing thus a minor percentage of our study cohort and an insufficient number to compare with.

This study aimed to investigate the clinical use of bleeding time in cohorts of TBI patients, as it is long performed in our unit. Therefore, correlation of the bleeding time with other coagulation parameters including platelet count is of importance since they both may reflect hemostasis. Nekludov et al. studied four patient groups (severe isolated TBI, general trauma without TBI, chronic alcohol abusers, and healthy volunteers). It was found that bleeding time in TBI patients was significantly prolonged compared with that in the other groups. In the same study, platelet count was similar in TBI patients and alcoholics [32]. Diagnosis and treatment of platelet dysfunction using platelet transfusion, platelet-stimulating drugs, or no treatment remain controversial in TBI. According to the current findings, AUD subjects had more prolonged values compared with those with no history of AUD. Moreover, the current results highlight possible difficulties in determining platelet function since an association between bleeding time and increased hematoma size was noted.

Someone can argue why subjects with markedly prolonged INR and PTT were excluded and why more specialized coagulation tests such as Thromboelastography (TEG), Rotational thromboelastography (ROTEM) and platelet mapping were not frequently carried out. The current study mainly investigated the potential added value of IVY bleeding time test when other coagulation abnormalities were in the normal range. Pathological INR and aPTT values would confuse the present findings and therefore, the conclusions on the value of the IVY bleeding time test. Moreover, these more specialized coagulation tests may provide more accurate data but also have their limitations (financial costs, sometimes time consuming, are not available in all hospitals); frequently, they cannot be done on a non-regular basis (during the night, weekends, etc.), and their interpretation requires special knowledge and training in the field compared with the easy interpreted results of bleeding time [26, 56]. Moreover, when compared with the common diagnostic tools (INR, PT, aPTT), fibrinolysis methods can be sometimes more cumbersome and less standardized [56]. Lastly, it can be postulated that alcohol may influence viscoelastic method parameters, possibly acting on platelets and especially in trauma patients [16].

Naturally, there is an association between platelet count and bleeding time. However, in the present study, this correlation was weak, since 46% of patients with prolonged bleeding time had platelet counts above 150,000/μL and 26% with normal bleeding time had low platelet counts. Following brain trauma, the bleeding time and other platelet functional tests may be prolonged, permanently or temporary, for some time secondary to the injury and irrespective of a normal platelet count. Whether this is due to a direct “platelet shock” by the brain trauma or secondary to other coagulation pathology, it remains debated. The different prevalence of a long bleeding time between the control and the AUD group can be related to multiple factors such as alcohol misuse, trauma severity, initial taking care, or medication, although this is difficult to state and is something which remains to be validated. The present findings can stimulate further studies to investigate whether dysfunctional platelets are of direct importance and not just an association with the outcome in TBI patients and if platelet transfusion can have a different effect (either positive or negative) on congenital platelet dysfunction or drug-induced platelet inhibition than in TBI.

We consider the IVY test to be primarily a test of platelet function/primary hemostasis and not of general coagulopathy. When a patient presents with a clearly elevated INR at 1.5 or higher, the IVY test would not routinely be employed in our department, in view of other important measures used to correct such coagulopathy (such as prothrombin complex concentrates). Regarding INR and TBI, although we acknowledge the fact that many recommend values > 1.2 as a limit for warfarin reversal, others suggest a target value of INR < 1.5 in cases with a positive CT scan [54]. Moreover, in surgical candidates, as TBI patients frequently are, an INR value of 1.5 or lower is commonly considered a limit where surgery can proceed safely [50, 54]. Of note, in the current study, no patient had an INR ≥ 1.5, only two had an INR of 1.4, and two had an INR of 1.3 on initial testing, representing thus a small percentage of the total cohort.

The study has limitations. Data were collected retrospectively. Information on the history of AUD was extracted from the patient archives and ICD codes, and no strict criteria or dedicated questionnaires could be applied mainly because of their critical condition. There was no available information on blood ethanol levels at the time of injury. Detailed clinical data and management data were in some occasions incomplete. Specialized coagulation tests to confirm or rule out bleeding tendency and tests to evaluate platelet function were typically not carried out. Since IVY has been used in a standardized way for a long time and performed by well-trained and experienced personnel in our unit with minor problems, we believe the commonly described variability of the IVY method in our unit is very limited. The exact time frame from injury to subjection to the IVY bleeding time test was not available for every individual. However, since the bleeding time test was available 24/7, it was typically performed not later than 8–12 h from injury. Due to small patient sample, multivariate analysis was not done. Further detailed data on the control group was not available. Lastly, clinical outcome at 6 months was available for 82.7% of patients.

## Conclusions

Although differences in the IVY bleeding time values between TBI cohorts exist and prolonged bleeding time values may be seen even in patients with normal platelet count, the bleeding test is a marker of platelet function and primary hemostasis with low specificity. Therefore, at this point, any specific recommendations for its use cannot be provided. However, the bleeding time test may provide an additional assessment in the interpretation of the overall status of the TBI patients with AUD. This test should be used as an indicator of primary hemostasis dysfunction in comparison with other cohorts only in combination with the patient’s bleeding history and careful assessment of other hematologic parameters.
